# Acute Femoropopliteal Bypass Graft Occlusion After Knee Manipulation Under Anesthesia: A Case Report and Review of Current Literature

**DOI:** 10.5435/JAAOSGlobal-D-21-00197

**Published:** 2022-02-02

**Authors:** Zachary W. Fulton, Amy Singleton, Kirk R. Davis

**Affiliations:** From the Mercy St. Vincent Medical Center, Toledo, OH.

## Abstract

Bilateral tibiofemoral knee dislocations are a relatively rare injury, and there is a scarcity of literature on its appropriate evaluation and treatment. Even less knee dislocations with concomitant popliteal artery injury have been described. Postoperative graft occlusion accounts for approximately half of the overall complication rate, occurring in up to 18% of the patients undergoing femoropopliteal bypass grafting. Furthermore, anticoagulation and antiplatelet therapy after graft placement is a point of contention. Here, we describe a case of a knee dislocation with associated popliteal artery transection treated initially with successful knee-spanning external fixation and arterial grafting, respectively. At 6 weeks after injury, the patient underwent external fixation removal and closed manipulation of the knee for arthrofibrosis. After manipulation, yet still under anesthesia, distal pulses were acutely diminished and subsequent CTA demonstrated femoropopliteal graft thrombosis. This case demonstrates successful recognition, thrombectomy, and restoration of arterial blood flow, which has since been maintained. Written consent by the patient involved in this case report was obtained.

Tibiofemoral knee dislocations (KDs) are rare, so recognition and treatment of associated arterial injury can be difficult.^1,2,3,4^ In addition, no gold standard for treating these concomitant orthopaedic and arterial injuries has been defined.^5^ KDs are reported as less than 0.02% of all orthopaedic injuries.^6^ Although there have been reported cases of bilateral KDs, there have been even fewer KDs with a concomitant popliteal artery injury.^1,2,3,4^ In 2018, Moura et al^6^ described a case of bilateral KDs with associated bilateral popliteal artery injuries, which was the first reported case at the time. A case series of two patients who required popliteal artery grafts after unilateral KDs reported limb survival; in their literature review, they found both a lower proportion of concomitant vascular injury with KDs and an even lower proportion that undergo surgical treatment than previously reported.^7^

In the acute setting, evaluation and treatment must be done rapidly to decrease the risk of limb ischemia.^8-10^ Reportedly, up to 20% of the patients presenting with KDs develop compartment syndrome and/or require limb amputation.^11^ The popliteal artery is the most frequently injured vessel in KDs, particularly posterior dislocations, with reported incidences varying from 20% to 40%.^6,12^ The best diagnostic approach for the assessment of vascular injury remains under review. A palpable dorsalis pedis (DP) pulse and posterior tibial (PT) pulse with an ankle-brachial index > 0.9 have been reported to have a sensitivity of 100% for excluding arterial injury.^13,14^ Routine computed tomography angiography (CTA) may not be the most suitable imaging modality depending on clinical findings and the medical center's available resources.^14,15^

Although the treatment of KDs is still controversial, the use of knee-spanning external fixation (ex-fix) for KDs has been accepted as a conservative mode of treatment, particularly in the setting of arterial injury in which the vascular team needs access to the extremity.^13,16^ Gross tibiofemoral instability may be treated with an ex-fix followed by rehabilitation, although immobilization should last < 6 weeks to avoid increased stiffness.^17^ Bodendorfer et al^18^ showed that the use of a knee-spanning ex-fix is a notable risk factor for arthrofibrosis after ligamentous knee injury. They also showed that early knee manipulation under anesthesia (MUA) leads to a more satisfactory outcome. MUA for arthrofibrosis has been noted as a safe procedure that successfully increases patient's range of motion (ROM).^19,20^

Acute femoropopliteal graft thrombosis after knee-spanning ex-fix removal and MUA, to our knowledge, has not been described in the literature. Here, we describe successful recognition, reperfusion, and limb salvage in a single patient at a level 1 trauma center. The patient's radiographic and angiographic imaging, treatment course, and outcome are also reported.

## Case Report

A 33-year-old male laborer with a history of substance abuse and psychiatric disorder was involved in an auto-pedestrian accident where he sustained bilateral KDs, a left hip dislocation, and a right clavicle fracture. On presentation, the patient's DP and PT pulses were absent bilaterally indicating possible arterial injury. His bilateral knees and left hip were reduced emergently with return of pulses to the left lower extremity but not the right. Ankle-brachial index was 0.62 on the left and unattainable on the right.

**Figure 1:**
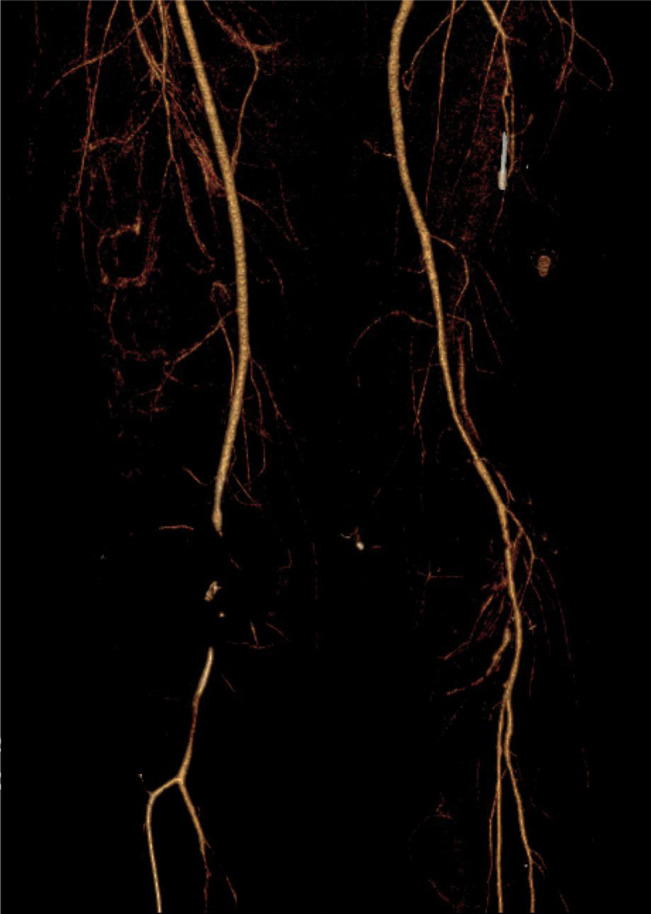
3D reconstruction of CTA demonstrating impaired runoff beginning at the proximal popliteal artery. CT image obtained at initial injury presentation.

**Figure 2:**
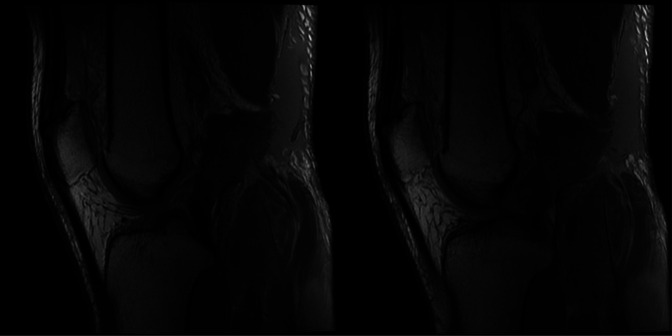
T1-weighted MRI of the right knee demonstrating complete rupture of anterior cruciate ligament and posterior cruciate ligament with associated gastrocnemius avulsion, indicative of an unstable tibiofemoral knee dislocation. MRI images obtained after the placement of knee-spanning external fixation.

CTA demonstrated a right popliteal artery transection and a left popliteal artery dissection with an associated intramural flap (Figure 1). The patient was taken to the operating room for emergent exploration of the right popliteal artery by vascular surgery and bilateral ex-fix placement by orthopaedics for gross tibiofemoral instability (Figure 2). Intraoperatively, complete popliteal artery transection was found on the right, and an Artegraft Collagen Vascular Graft (LeMaitre Vascular) was used as a femoropopliteal bypass followed by four-compartment fasciotomies. Postoperatively, the patient had palpable DP and PT pulses bilaterally, confirmed by doppler. No acute complications were observed after the initial surgery, and he was started on an antiplatelet regimen of aspirin 81 mg daily. Vascular surgery did not recommend surgical intervention for the left popliteal artery injury. The patient was scheduled for bilateral ex-fix removal with knee MUA at 6 weeks postoperatively, followed by physical therapy to regain his ROM.

**Figure 3:**
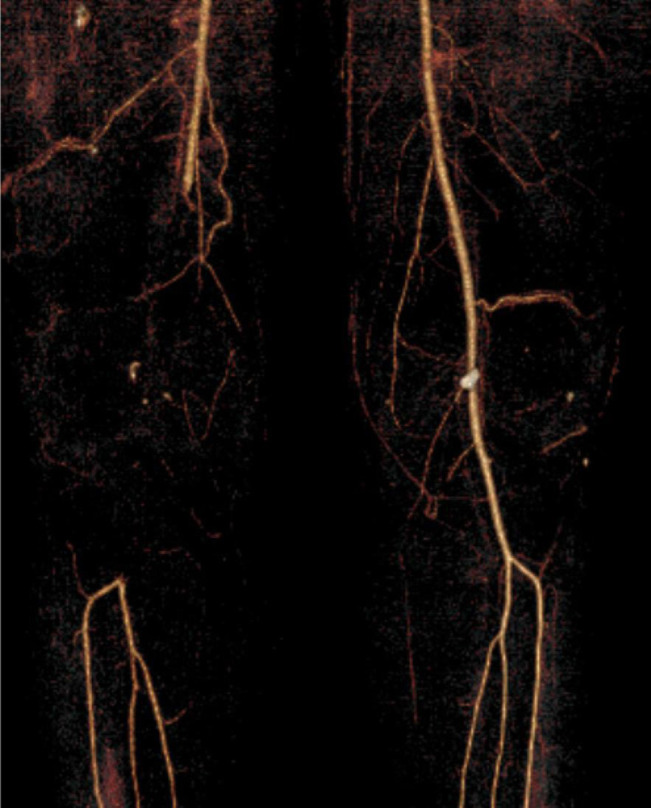
3D reconstruction of CTA demonstrating complete graft thrombosis in the right lower extremity. CT image obtained after external fixation removal and manipulation under anesthesia.

**Figure 4:**
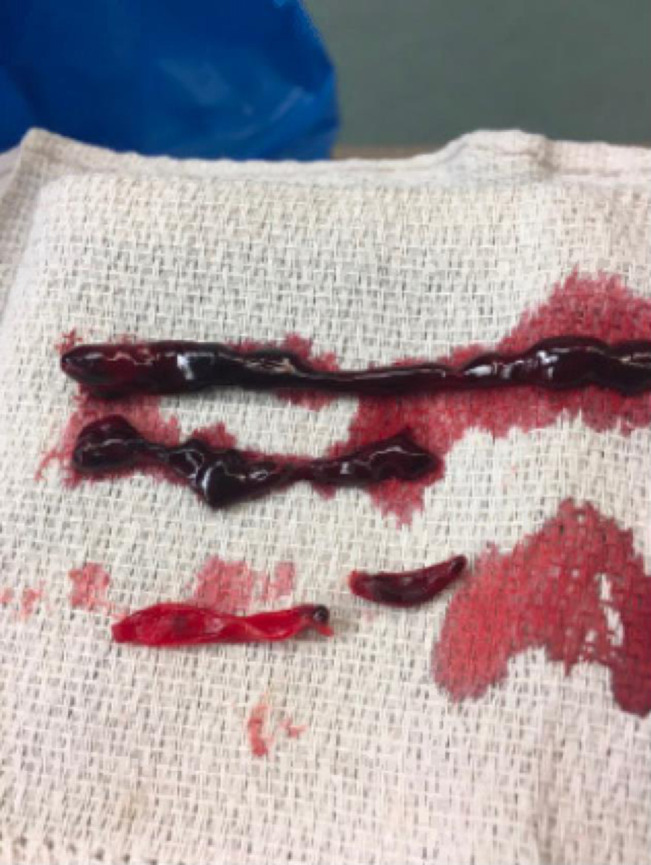
Clinical photograph of the thrombus that was removed from the right femoropopliteal bypass graft during the thrombectomy procedure. Images obtained during the procedure.

Two days before the scheduled ex-fix removal, a vascular surgeon assessed this patient and noted bilateral palpable DP and PT pulses. The patient also had orthopaedic preoperative evaluation on the day of surgery that demonstrated 2 + DP and PT pulses. Ex-fix removal proceeded without complication, and intraoperative knee passive ROM (PROM) was assessed and recorded as zero to 30° bilaterally. Both knees were manipulated using a short lever arm technique until the scar tissue was released. Final knee PROM improved to zero to 105° bilaterally. DP and PT pulses were reassessed and were nonpalpable on the right. Using doppler, the graft flow was minimally, but audibly present. In recovery, duplex ultrasonography of the right DP and PT arteries showed inadequate flow; subsequent CTA demonstrated thrombosis of the right femoropopliteal bypass graft (Figure 3). The patient underwent emergent thrombectomy and revascularization with the successful removal of a large thrombus from the graft site (Figure 4). Postoperatively, he was placed on a heparin drip and was ultimately discharged on an anticoagulation regimen of apixaban 2.5 mg twice daily and aspirin (ASA) 81 mg daily.

At the patient's 5-week follow-up, there were no complaints of instability or joint line pain. Active knee ROM was 10° to 75° and 10° to 80° for the left and right knees, respectively. His DP and PT pulses were palpable bilaterally. The patient was ambulating independently and planned to continue working with physical therapy to increase his ROM and muscle strength. The patient was then lost to orthopaedic follow-up after 5 weeks postoperatively, and the last documented vascular status was 4 months postoperatively in the emergency department, which noted palpable lower extremity pulses and no other related report.

## Discussion

The successful treatment of concomitant KDs and arterial compromise highlights the need for interdisciplinary teamwork for a successful patient outcome. Femoropopliteal bypass grafting has an overall 30-day morbidity rate of 37%.^5^ Postoperative graft occlusion accounts for approximately half of the overall rate, occurring in up to 18% of the patients. An analysis of graft patency after manipulation procedures has not been previously reported in the literature. Femoropopliteal graft patency relies on several factors, including graft material and length.^21-23^ A biologic bovine carotid graft was used for a femoropopliteal bypass in this patient. The optimal graft type for lower extremity bypass continues to be an area of discussion, with autologous generally being the preferred choice when available, and bovine carotid, an acceptable substitute, that does not markedly compromise limb salvage or patency in urgent situations where minimal dissection and surgical time are imperative.^24,25,26,27^

Antiplatelet therapy with femoropopliteal grafting is another point of consideration in this case; no standard protocol is currently accepted.^24,28^ Approximately 10% of the femoropopliteal and femorodistal grafts fail within 1 month postoperatively while most of the grafts (80%) fail between 1 month and 2 years, requiring surgical reintervention.^29^ In a large systematic review of antiplatelet therapy in the use of preventing thrombosis in patients undergoing femoropopliteal grafting, Brown et al^30^ found that ASA or ASA with dipyridamole had a beneficial effect on primary patency of the grafts. The specific duration and timing of ASA therapy for patients needing revascularization procedures are still uncertain.^31^ In a study comparing ASA with ASA with warfarin therapy, no benefit was found to adding warfarin, but there was a large increase in morbidity and mortality with its use.^32^

In this case, the timing of the change in arterial physical examination preoperatively to postoperatively is highly suggestive of an intraoperative event causing a reduction in arterial flow to the foot. These events include graft thrombosis, graft transection, or collateral arterial injury in the setting of a chronically occluded graft site. Although possible, it is unlikely that the patient had a chronic graft occlusion before MUA. No preoperative angiogram was done that may have changed the postoperative course had there been a prior chronic occlusion. A clot removed from the graft in the setting of acute loss in distal pulses suggests that there was an intraoperative event rather than a chronic one found in the postanesthesia care unit (PACU).

MUA is a treatment used in a wide array of situations, including knee arthrofibrosis, posttotal joint arthroplasty stiffness, and adhesive capsulitis.^33-35^ For this patient, a short lever arm technique was done, as opposed to the long lever arm technique, which has been shown to have a higher rate of complications, which can include fracture and the need for revision surgery.^19^ MUA for arthrofibrosis has been believed as a relatively benign, minimally invasive procedure to increase a patient's ROM, but here we show that there can be consequences for certain at-risk patients, and even potentially lead to limb loss.^19,20^ Preparedness was a key driver of preventing limb loss in this patient; as a medical community, there is still room for improvement in understanding the contraindications and prevention of complications for knee MUA after femoropopliteal bypass grafting.^33^

## Conclusion

Few reports of bilateral KDs and fewer with vascular complications requiring arterial bypass or repair can be found in the literature. Limited knowledge on the risk of complications for knee MUA after arterial repair exists. Here, we describe a rare case of femoropopliteal graft thrombosis occurring during knee MUA, with successful outcome after interdisciplinary intervention.
